# Increased levels of a pro-inflammatory IgG receptor in the midbrain of people with schizophrenia

**DOI:** 10.1186/s12974-022-02541-8

**Published:** 2022-07-15

**Authors:** A. Petty, L. J. Glass, D. A. Rothmond, T. Purves-Tyson, A. Sweeney, Y. Kondo, S. Kubo, M. Matsumoto, C. Shannon Weickert

**Affiliations:** 1 Schizophrenia Research Laboratory, Neuroscience Research Australia, Sydney, NSW 2031 Australia; 2 School of Psychiatry, University of New South Wales, Sydney, NSW 2052 Australia; 3 Centre for Immunology and Allergy Research, Westmead Institute of Medical Research, The University of Sydney, Sydney, Australia; 4 NSW Brain Tissue Resource Centre, University of Sydney, Sydney, NSW 2006 Australia; 5 Astellas Research Institute of America LLC, San Diego, CA 92121 USA; 6 Astellas Pharma Inc., Tsukuba, Ibaraki 305-8585 Japan; 7 Department of Neuroscience and Physiology, Upstate Medical University, Syracuse, NY 13210 USA

**Keywords:** Schizophrenia, Inflammation, Midbrain, Antibodies, Antibody receptor, FcGR3A, Post-mortem

## Abstract

**Background:**

There is growing evidence that neuroinflammation may contribute to schizophrenia neuropathology. Elevated pro-inflammatory cytokines are evident in the midbrain from schizophrenia subjects, findings that are driven by a subgroup of patients, characterised as a “high inflammation” biotype. Cytokines trigger the release of antibodies, of which immunoglobulin G (IgG) is the most common. The level and function of IgG is regulated by its transporter (FcGRT) and by pro-inflammatory IgG receptors (including FcGR3A) in balance with the anti-inflammatory IgG receptor FcGR2B. Testing whether abnormalities in IgG activity contribute to the neuroinflammatory abnormalities schizophrenia patients, particularly those with elevated cytokines, may help identify novel treatment targets.

**Methods:**

Post-mortem midbrain tissue from healthy controls and schizophrenia cases (*n* = 58 total) was used to determine the localisation and abundance of IgG and IgG transporters and receptors in the midbrain of healthy controls and schizophrenia patients. Protein levels of IgG and FcGRT were quantified using western blot, and gene transcript levels of FcGRT, FcGR3A and FcGR2B were assessed using qPCR. The distribution of IgG in the midbrain was assessed using immunohistochemistry and immunofluorescence. Results were compared between diagnostic (schizophrenia vs control) and inflammatory (high vs low inflammation) groups.

**Results:**

We found that IgG and FcGRT protein abundance (relative to β-actin) was unchanged in people with schizophrenia compared with controls irrespective of inflammatory subtype. In contrast, FcGRT and FcGR3A mRNA levels were elevated in the midbrain from “high inflammation” schizophrenia cases (FcGRT; *p* = 0.02, FcGR3A; *p* < 0.0001) in comparison to low-inflammation patients and healthy controls, while FcGR2B mRNA levels were unchanged. IgG immunoreactivity was evident in the midbrain, and approximately 24% of all individuals (control subjects and schizophrenia cases) showed diffusion of IgG from blood vessels into the brain. However, the intensity and distribution of IgG was comparable across schizophrenia cases and control subjects.

**Conclusion:**

These findings suggest that an increase in the pro-inflammatory Fcγ receptor FcGR3A, rather than an overall increase in IgG levels, contribute to midbrain neuroinflammation in schizophrenia patients. However, more precise information about IgG-Fcγ receptor interactions is needed to determine their potential role in schizophrenia neuropathology.

**Supplementary Information:**

The online version contains supplementary material available at 10.1186/s12974-022-02541-8.

## Introduction

Growing evidence from post-mortem brain and blood biomarker studies suggests that neuroinflammation is a feature of schizophrenia [1–8]. Specifically, there is now evidence of significantly increased levels of pro-inflammatory cytokines—small signalling proteins within the immune system—in the midbrain of patients with schizophrenia [5]. Furthermore, this finding is driven by a subgroup of “high inflammation” patients, identified using a two-step recursive clustering algorithm with the following input transcripts; interleukin (IL)-1β, IL-6, tumour necrosis factor (TNF)-α, and acute phase protein serpin peptidase inhibitor clade A member 3 (SERPINA3), from the midbrain [5]. This same clustering technique has also been applied to cytokine levels in other brain regions, and reveals a similar clustering of low- and high-inflammation biotype schizophrenia patients [3, 4, 9, 10]. As well as these cytokines, previous work has found other inflammation-mediating factors to be increased in the midbrain of high-inflammation patients with schizophrenia. These include the intracellular adhesion molecule (ICAM1) [11] which is instrumental in the migration of B cells, T cells, and monocytes into the brain [12–16]. Previous studies have also found increased levels of multiple complement cascade factors, as well as increased macrophage, microglia and astrocyte cell markers in the midbrain of high-inflammation schizophrenia patients [11]. Activation of the classical complement pathway (and other inflammatory processes) relies on antibody binding. However, the abundance and distribution of immunoglobulin gamma or γ (IgG; the most common antibody circulating in the blood) and its transporter and receptors has not been explored in the midbrain of schizophrenia patients.

Given the increase in pro-inflammatory cytokines in the midbrain, we predict elevated antibody levels in the high-inflammation schizophrenia subgroup in this brain region. IgG may enter the brain from the microvasculature which pervades the brain tissue. Additionally, B cells, which generate antibodies, may themselves enter the brain and generate IgG locally. The blood–brain barrier normally prevents the movement of large molecules and cells from the periphery into the central nervous system. However, B cells have been found in the brain parenchyma of healthy people [17] and in subsets of people with schizophrenia [18]. Recent studies also suggest that the meninges which surround the brain may represent a source of locally generated B cells which infiltrate the brain [19]. There is also evidence that macrophages enter the brain parenchyma in experimental studies in rodents following stress [20], and possibly in schizophrenia [5, 8, 16]. Change in the blood brain barrier due to inflammation may facilitate the entry of IgG [or IgG-producing B cells [21]] into the midbrain of patients with schizophrenia. Therefore, we predict elevated IgG levels in the midbrain of people with schizophrenia, particularly in the high-inflammation subgroup.

**Fig. 1:**
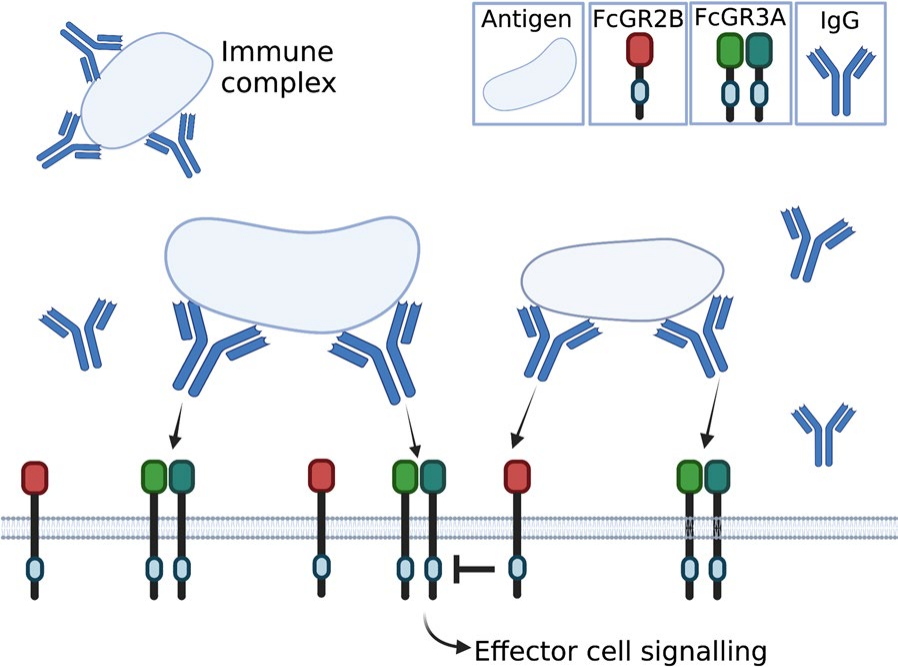
Diagram of the action of IgG and its receptors. When IgG binds to an antigen, it forms an immune complex. This antigen may be a foreign pathogen (e.g. a bacteria or virus) but may also be an autoantigen  generated within the individual. The immune complex binds to IgG receptors via the Fc region of the IgG antibody. When the immune complex binds to FcGR3A, the immunoreceptor tyrosine-based activating motif (ITAM) is phosphorylated, and a signalling cascade initiates the engulfment/degradation of the antigen. This pro-inflammatory effect is balanced by the anti-inflammatory Fc receptor, FcGR2B. When an immune complex binds to FcGR2B, the immunoreceptor tyrosine inhibitory motif (ITIM) is phosphorylated to suppress this signalling cascade therefore terminating the immune response (not to scale)

Baseline levels of IgG are mediated by its transporter, and the function of IgG is modulated by IgG receptors located on effector cells. The Fcγ receptor and transporter—FcγRT (also known as the neonatal Fc receptor) binds to IgG, and can sort it into recycling endosomes [22], before returning it to the cell surface for release into the extracellular space [23, 24]. This mechanism maintains healthy IgG concentrations throughout the body [25]. Once IgG has bound to an antigen (forming an immune complex), it then binds to an IgG Fc receptor (called Fcγ or FcG receptors) through its Fc region (Fig. 1). These receptors are expressed on the membrane of a range of cells including macrophages, microglia and astrocytes. Most Fcƴ receptors are pro-inflammatory; when the immune complex binds to the Fcƴ receptor, the immunoreceptor tyrosine-based activating motif (ITAM) is phosphorylated [26, 27], and a signalling cascade triggers phagocytosis [28, 29] and cytotoxic granule release [30–32]. Increased mRNA levels of the pro-inflammatory receptor FcGR3A (also called CD16a) have already been found in the prefrontal cortex [16] and the subependymal zone [9] of high-inflammation schizophrenia cases, and may reflect an increase in infiltration of, for instance, macrophages. We will therefore determine whether FcGR3A mRNA is also increased in the midbrain. The actions of pro-inflammatory Fcƴ receptors are balanced by the anti-inflammatory Fcƴ receptor FcGR2B (also known as CD32b). FcGR2B has a cytoplasmic immunoreceptor tyrosine *inhibitory* motif (ITIM) [33] which is phosphorylated upon IgG binding. FcGR2B then suppresses signalling initiated by phosphorylated ITAM of pro-inflammatory Fcy receptors like FcGR3A, thereby terminating the immune response [34, 35]. This balance of FcGR2B and pro-inflammatory receptors is critical for re-establishing homeostasis after an immune response. Levels of FcGR2B mRNA have not been determined in the brains of people with schizophrenia.

The primary aims of this study were therefore to determine (a) whether protein and/or mRNA levels of IgG, its transporter, or IgG receptors were altered in the midbrain of schizophrenia cases compared to control subjects, and; (b) whether levels of IgG protein, or IgG transporter or IgG receptor transcripts differed when comparing high-inflammation and low-inflammation subtypes of schizophrenia cases. We further sought to determine whether the localisation of midbrain IgG immunoreactivity was different when comparing diagnostic groups.

## Methods

### Cohort of midbrain tissue from schizophrenia cases and control subjects

The use of post-mortem human brain tissue was approved by the University of New South Wales Human Research Ethics Committee (HC17826). Post-mortem midbrain samples (*N* = 58 individuals) were obtained from the New South Wales Brain Tissue Resource Centre. Two overlapping cohorts were used in this study; one for tissue homogenate analyses (mRNA and protein; Table 1) and one for 3,3′-diaminobenzidine (DAB) immunohistochemistry (Table 2). Two schizophrenia subjects were omitted from the protein analysis due to poor protein quality, however this did not alter any key demographics. The case and control cohorts used in this study have been previously described [5, 36]. For the mRNA and protein analysis, schizophrenia cases were classified into high- or low-inflammation biotypes based on elevated IL-6, IL-1β, SERPINA3, and TNFα mRNA levels in the midbrain, using a two-step recursive clustering analysis [3]. Based on this clustering, all control subjects clustered into the low-inflammation biotype. The high-inflammation biotype was found in ~ 46% (*n* = 13) of schizophrenia cases. For the double-labelled immunofluorescence experiment, a subsidiary cohort was generated using 10 subjects from each subgroup (control, low inflammation, high inflammation; demographics in Additional file 1: Table S1). In all cohorts, diagnostic groups were matched for age, sex, and post-mortem interval (and RNA integrity number [RIN] where relevant). Significant differences in antipsychotic history were apparent for the tissue homogenate cohort and the immunofluorescence cohort when comparing the low and high-inflammation schizophrenia subgroups. High-inflammation schizophrenia patients had higher last recorded chlorpromazine (CPZ) equivalent dose (homogenate cohort; *p* = 0.01, immunofluorescence cohort; *p* = 0.03), and an increased daily CPZ equivalent dose (homogenate cohort; *p* = 0.01, immunofluorescence cohort; *p* = 0.05) in comparison to low-inflammation biotype subjects. The immunofluorescence cohort also showed an increased age at illness onset for the high-inflammation subgroup (*p* = 0.03).

**Table 1:** Demographic and clinical variables comparing control subjects with either all schizophrenia cases, or control subjects compared to both the low-inflammation and high-inflammation schizophrenia biotype groups, for the tissue homogenate analyses (mRNA and protein)

Demographics	Tissue homogenate cohort					
	Control	Schizophrenia	Statistics	Schizophrenia (low inflammation)	Schizophrenia (high inflammation)	Statistics
*n*	28	28		15	13	
Age (years)	52.5 (22–67)	54.5 (26–67)	*t*_(54)_ = − 0.27, *p* = 0.79	51.0 (30–64)	56.0 (26–67)	*F*_(2, 53)_ = − 1.24, *p* = 0.29
Sex (M,F)	19,9	19,9	*X*^*2*^ = 0.000, *p* = 1.0	11,4	8,5	*X*^*2*^ = 0.44, *p* = 0.80
pH	6.6 ± 0.26 (5.8–67.0)	6.5 ± 0.20 (6.14–6.88)	***t***_**(54)**_ = 2.52 *p* = 0.01^**^	6.5 ± 0.2 (6.14–6.88)	6.4 ± 0.2 (6.20–6.79)	*F*_(2, 53)_ = 3.12, *p* = 0.052
PMI (h)	31.6 ± 10.2 (15–50)	35.6 ± 17.7 (5–72)	*t*_(43.17)_ = − 1.03, *p* = 0.31	33.4 ± 14.7 (18–64)	38.2 ± 21.0 (5–72)	*F*_(2, 53)_ = − 0.86, *p* = 0.42
RIN	5.5 ± 1.1 (3.0–7.3)	5.6 ± 1.3 (3.2–8.3)	*t*_(54)_ = − 0.15, *p* = 0.88	5.5 ± 1.2 (3.30–7.20)	5.7 ± 1.3 (3.20–8.30)	*F*_(2, 53)_ = − 0.07, *p* = 0.92
Age at illness onset (years)	–	22.5 ± 7.4 (15–46)	–	21.5 ± 7.1 (15–39)	23.7 ± 7.9 (16–46)	
Duration of illness (years)	–	28.8 ± 12.7 (3.5–49)	–	26.4 ± 11.6 (3.5–43)	31.6 ± 13.9 (5–49)	*t*_(26)_ = − 1.07, *p* = 0.29
Daily CPZ equivalent dose (mg)^a^		736.4 ± 520.4 (200–2363)		483.5 ± 177.6 (200–800)	1040.1 ± 637.1 (250–2362)	***t***_**(20)**_** = **− 2.91, *p* = 0.01^**^
Last recorded CPZ equivalent dose (mg)^b^	–	597.5 ± 497.6 (20–1732)	–	361.5 ± 316.6 (20–1333)	869.9 ± 538.8 (100–1732)	***t***_***(*26)**_** = **− 3.09, *p* = 0.01^**^

Bold indicates statistical significance

Age is written as median (range). All other values are written as mean ± standard deviation (range). M = male, F = female; RIN = RNA integrity number; PMI = post-mortem interval; CPZ = chlorpromazine. ^a^ schizophrenia cases n = 19. ^b^ schizophrenia cases n = 25

**Table 2:** Demographic and clinical variables comparing control subjects with schizophrenia cases for the DAB immunohistochemical analysis

Demographics	DAB immunohistochemistry cohort		
	Control	Schizophrenia	Statistics
n	28	30	–
Age (years)	52.5 (22–69)	54.5 (26–67)	*t*_(56)_ = − 0.27, *p* = 0.78
Sex (M, F)	18,10	19,11	*X*^*2*^ = 0.006, *p* = 0.94
pH	6.6 ± 0.28 (5.8–7.0)	6.4 ± 0.23 (5.8–6.8)	***t***_**(56)**_ = 2.56 *p* = 0.01^**^
PMI (h)	32.8 ± 9.7 (15–50)	36.2 ± 17.7 (5–72)	*t*_(45.5)_ = − 0.90, *p* = 0.37
RIN	5.4 ± 1.2 (2.6–7.3)	5.4 ± 1.4 (2.7–8.3)	*t*_(56)_ = 0.14, *p* = 0.98
Age at illness onset (years)	–	22.2 ± 7.2 (15–46)	–
Duration of illness (years)	–	29.6 ± 12.7 (4–49)	–
Daily CPZ equivalent dose (mg)^a^	–	736.5 ± 520.5 (200–2363)	–
Last recorded CPZ equivalent dose (mg)^b^	–	630.7 ± 520.3 (20–1732)	–

Bold indicates statistical significance

Age is written as median (range). All other values are written as mean ± standard deviation (range). M = male, F = female; RIN = RNA integrity number; PMI = post-mortem interval; CPZ = chlorpromazine. ^a^ schizophrenia cases n = 19. ^b^ schizophrenia cases n = 25

When we examine the presence of systemic inflammation at the time of death, we see that inflammation was present for 5/28 (17%) controls, 4/15 (26%) low-inflammation schizophrenia, and 8/13 (61%) of high-inflammation schizophrenia. A Chi-square analysis revealed a significant difference between these groups for the evidence of body-wide inflammation at the time of death (*X* = 8.1, *p* = 0.017). This is a similar pattern to that found in a comparable study [3]. The two most common somatic comorbidities alongside schizophrenia were asthma and diabetes. When we compared the prevalence of asthma and diabetes between schizophrenia inflammatory subgroups, no difference was evident in a Chi-square analysis (*X* = 2.7, *p* = 0.09). There was also no difference in BMI between all groups (*F*_(2,50)_ = 0.48, *p* = 0.95), or between high and low-inflammation schizophrenia subgroups (*t*_(24)_ = − 3.06, *p* = 0.38). See Additional file 2: Table S2 for all demographic data from the cases used in this study.

### Western blot

Protein was extracted from fresh-frozen tissue, which had been dissected from 6 × 60 μm-thick coronal midbrain sections from each subject as previously described (36). Protein levels were quantified using a Bradford assay (B6916, Sigma), and 10 μg of midbrain protein was used in a Western blot to assess levels of IgG and FcGRT. Midbrain protein (in loading buffer of equal volume; Laemmli buffer (161–0737, Bio-Rad) with 2.5% β-mercaptoethanol) was heated at 95 °C for 5 min and loaded into 10% polyacrylamide gels alongside two molecular weight ladders (7.5 μl Chameoleon Duo Ladder, 928–60,000, LI-COR, Lincoln, NE, USA). Protein was separated by electrophoresis at 200 V for 50 min after which gels were equilibrated in running buffer for 10 min. Protein was transferred from gel to methanol submerged PVDF membrane (Immobilon-FL PVDF, IPFL20200, Merck Millipore) for 1.5 h at 100 V in transfer buffer (0.14 M glycine, 18.6 mM Tris, 20% methanol in deionised water). Membranes were washed three times for 5 min in TBS with 0.01% Tween-20 while rocking before 1 h incubation in blocking buffer [1:1 Odyssey blocking buffer (927–50,000, LI-COR)] in TBS.

The mouse anti-β-actin primary (1:10,000; MAB1501, Merck Millipore, Billerica, MA, USA) was used as the loading control. To probe for FcGRT, this was combined with rabbit anti-FcGRT (1:200; Sc-66892, Santa Cruz), diluted in blocking buffer with 0.01% Tween-20 and applied to the membranes. The following day membranes were drained and washed three times in TBS with 0.05% Tween-20. The secondaries used to detect β-actin and FcGRT were IRDye® 680CW conjugated donkey anti-mouse (1:10,000; 927-4, LI-Cor) and IRDye® 800CW donkey anti-rabbit (1:15,000; 925–999,999, LI-COR), respectively. To detect IgG, mouse anti-β-actin (1:10,000; MAB1501, Merck Millipore, Billerica, MA, USA) was used alone as the primary antibody using the same protocol as above. On the following day, membranes were treated as above. Then IRDye® 680CW conjugated donkey anti-mouse (1:10,000; 927–4, LI-Cor) was used to detect β-actin and IRDye® 800CW conjugated goat anti-human IgG (1:15,000; 926–32,232, LI-COR) was used to detect IgG. Membranes were incubated with the secondary antibody for 1.5 h at RT while slowly rocking. Membranes were washed two times in TBS and once in deionised water before imaging with the Odyssey Clx scanner (LI-COR). Background florescence was subtracted from the band of interest by a user-defined region of interest. Immunoreactive band intensity of the region of interest was normalised to β-actin band intensity in the same lane and then to the internal control from the same membrane. No significant differences were found when comparing β-actin protein levels in the midbrain from schizophrenia cases and control subjects (*p* > 0.05). For representative gel images, please see Additional file 3.

### Analysing gene transcript levels with qPCR

Trizol was used to extract total RNA from schizophrenia and control midbrain tissue, and cDNA was synthesised with Superscript III (Life Technologies, Scoresby, Australia) as described previously [36, 37]. Only cases whose RNA integrity number (RIN) was > 4.0 were included in the analysis. FcGRT, FcGR2B, FcGR3A mRNA was measured by quantitative PCR (qPCR) using Applied Biosystem Prism 7900HT Fast Real Time system and predesigned TaqMan assays listed in Additional file 1: Table S3. In all cases, mRNA was normalised to the geomean of expression levels of three housekeeper genes; β-actin, TATA binding protein, ubiquitin-C, which did not differ between schizophrenia cases and control subjects (*t*_(55)_ = 0.74, *p* > 0.05).

### DAB immunohistochemistry

14 μm sections (adjacent to the tissue used for homogenates above) of fresh-frozen midbrain were thawed to room temperature (23 °C, RT) and fixed in 4% paraformaldehyde for 10 min. Endogenous peroxides were quenched by washing in 3:1 methanol with 3% hydrogen peroxide for 20 min. Slides were incubated overnight with 10% normal blocking serum [1:10 normal goat serum (Vector Laboratories) in diluent (0.3% Triton X-100 and 0.05% bovine serum albumin in PBS)] in a humidified chamber at 4 °C. The following morning, slides were drained of serum, and biotinylated goat anti-human IgG preabsorbed against chicken, cow, horse, mouse, pig, rabbit, and rat (1:250; Ab97168, Abcam) in diluent was applied to the sections for a 1-h incubation in a humidified chamber at RT. Slides were washed three times for 5 min in PBS while rocking and avidin–biotin-peroxidase complex (ABC, VectaStain ABC kit, PK-4000, Vector Laboratories Peterborough, UK) was applied for a 1 h humidified incubation at RT. ABC was drained from the slides and slides were washed another three times in PBS before 250 μl DAB (12 mM DAB in PBS with 3% H_2_O_2_) was applied for ~ 2.5 min. The slides were Nissl counterstaining in 0.02% thionine. Sections were cleared with xylene before cover slipping with permount (Thermo Fisher Scientific). Slides were analysed by brightfield microscopy and imaged with StereoInvestigator using a Nikon Eclipse i80 at 20 × magnification (Nikon, Tokyo, Japan).

### Double-label immunofluorescence

The localisation of IgG and tyrosine hydroxylase (TH; a marker for dopamine neurons) in the midbrain was visualised using double-labelled immunofluorescence. 14 μm midbrain sections from schizophrenia cases and control subjects were thawed at RT as above, and then fixed in 4% paraformaldehyde for 10 min. Sections were incubated with normal donkey serum (1:10, Jackson Laboratories, Baltimore, MD, USA) and normal goat serum (1:10, Vector Laboratories) in diluent (0.3% Triton X-100 and 0.05% bovine serum albumin in PBS) for 1 h at RT. The primary antibody solution included mouse anti-TH (1:400, MAB318, Millipore) to label dopaminergic neurons, together with the same biotinylated goat anti-human IgG as described above (1:100, AB97168, Abcam) to label IgG. Two sections from each subject were labelled with both antibodies, and a third section (no-IgG) was labelled with only the TH antibody (omitting the IgG antibody). The antibody solution was left on overnight in a humidified chamber at 4˚C. The following day sections were incubated in the dark for 1 h at RT with the following proteins in diluent: donkey anti-mouse AlexaFluor488 preabsorbed against chicken, cow, goat, human, rabbit, rat, and sheep (1:500, Ab1050109, Abcam), and goat AlexaFluor647 conjugated streptavidin (1:1000, S21374, Invitrogen, Eugene, OR, USA). Sections were counterstained with DAPI (1:1000; Sigma-Aldrich). Tissue was washed in a 5 mM cupric sulfate and 50 mM ammonium acetate solution to quench autofluorescence. After final washes, sections were mounted with Citifluor anti-fade mounting solution (IAF1, ProSci Tech, Thuringowa, QLD, Australia).

Images were acquired with a LSM800 Zeiss confocal microscope (Zeiss Australia, Lonsdale, AUS), using a 20 × air objective. 15 snapshots (“images”) of 320 × 320 um were taken throughout the SN (indicated by TH + cells) for sections labelled with TH and IgG, and 3 images were taken for those only labelled with TH. The mean intensity value (mean gray value) of the fluorescent signal for the 647 (IgG) channel was measured using ImageJ. To account for any differences between individuals in non-specific binding of the streptavidin label, the delta gray value for IgG was also determined; the fluorescent signal of the no-IgG section was subtracted from the fluorescent signal of the IgG-stained section for each individual using the same imaging parameters. Additionally, an automatic threshold was applied to the double-labelled images to determine the areas of high IgG intensity, and the size of these areas was quantified using the “analyse particles” function of ImageJ (see Additional file 1: Figure S1 for the pipeline).

### Statistical analysis

Statistical analysis was performed using SPSS (ver. 24). Normality was determined by Shapiro–Wilk’s test (*p* > 0.05) and outliers were identified by an iterative Grubb’s test and removed (see Additional file 1: Table S4 for outliers removed). Where necessary, normality was achieved by logarithmic transformation. Pearson’s correlation coefficient or Spearman’s Rho (for non-normal data) were used to determine correlation between demographic or clinical variables and experimental measures. Student’s *t*-test or Mann–Whitney *U* tests were used for parametric or non-parametric comparisons between schizophrenia cases and control subjects. Analysis of variance (ANOVA or ANCOVAs) were used for comparisons between high-inflammation schizophrenia cases, low-inflammation schizophrenia cases, and control subjects.

## Results

### The abundance of IgG and FcGRT protein was not significantly altered in the midbrain from schizophrenia cases compared to control subjects

**Fig. 2:**
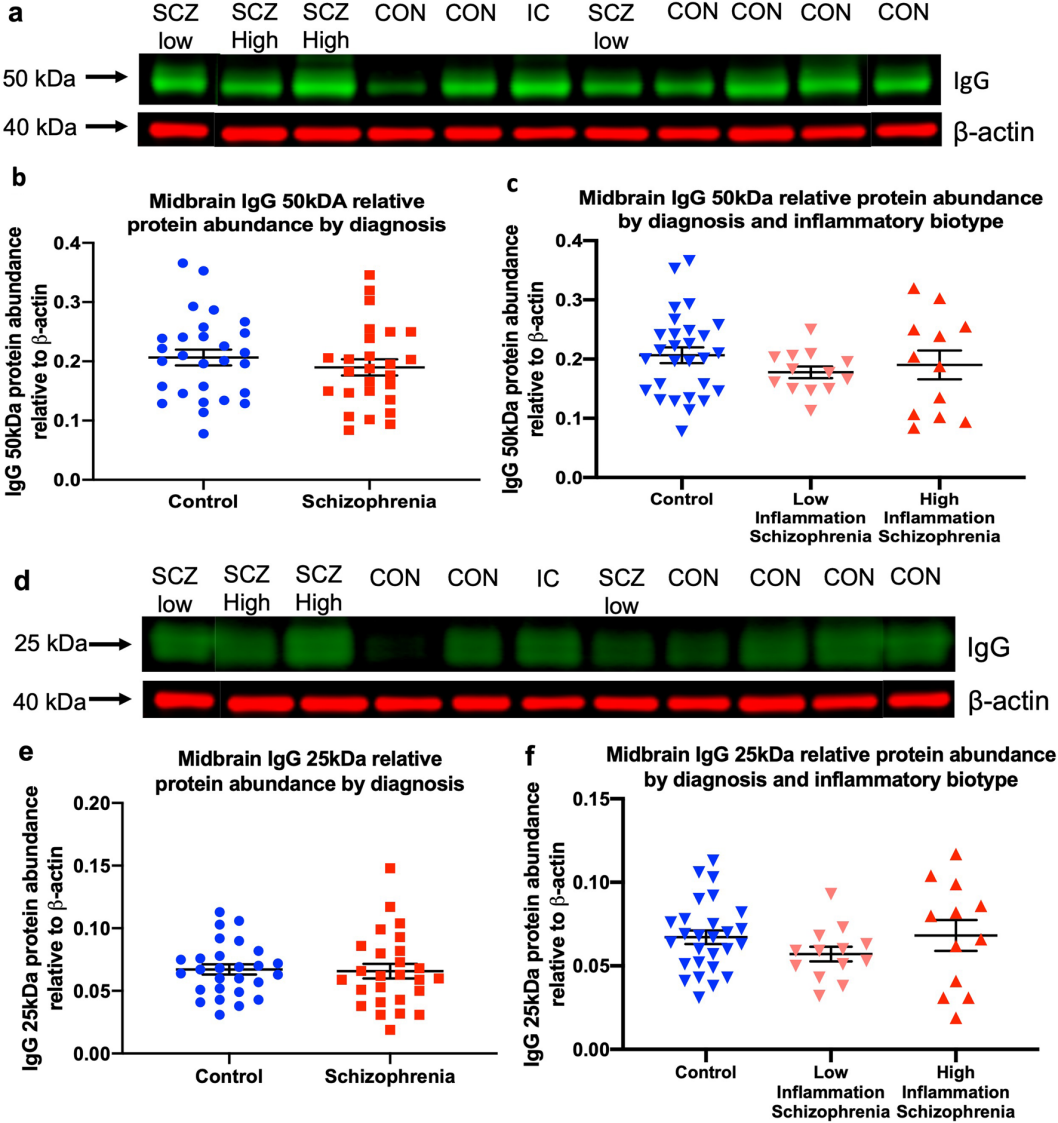
IgG 50kDA (**a**–**c**) and 25-kDa (**d**–**f**) protein abundance was comparable between schizophrenia cases and control subjects. IgG 50-kDa and 25-kDa immunoreactive bands were detected in midbrain protein homogenates from all individuals. IgG protein abundance relative to β-actin was unchanged when comparing midbrain from schizophrenia cases (**b**, **e**) and control subjects (**b**, **e**). No significant differences were found when comparing midbrain IgG protein abundance from high-inflammation biotype schizophrenia cases, low-inflammation biotype schizophrenia cases, and control subjects (**c**, **f**). kDa; kilodalton, CON; control subject, IC; internal control, SCZ Low; low-inflammation schizophrenia case, SCZ High; high-inflammation schizophrenia case. Mean ± SEM

The abundance of IgG immunoreactive bands (50 kDa; Fig. 1 and 25 kDa; Fig. 2d) relative to β-actin was unchanged in the midbrain from schizophrenia cases when compared to control subjects (IgG 50 kDa: *t*_(52)_ = 0.88, *p* = 0.39; IgG 25 kDa: *t*_(51)_ = 0.21, *p* = 0.83; Fig. 2b and e). Additionally, IgG 50 kDa and 25 kDa relative immunoreactivity was comparable when inflammatory biotype of schizophrenia cases was taken into account (IgG 50 kDa: *F*_(2,46)_ = 0.87, *p* = 0.42; IgG 25KDa: *F*_(2,47)_ = 0.09, *p* = 0.92; Fig. 2c and f). Comparable β-actin abundance was found in midbrain from schizophrenia cases and control subjects across the 2 rounds of testing performed (Round 1, *p* = 0.99; Round 2, *p* = 0.28).

**Fig. 3:**
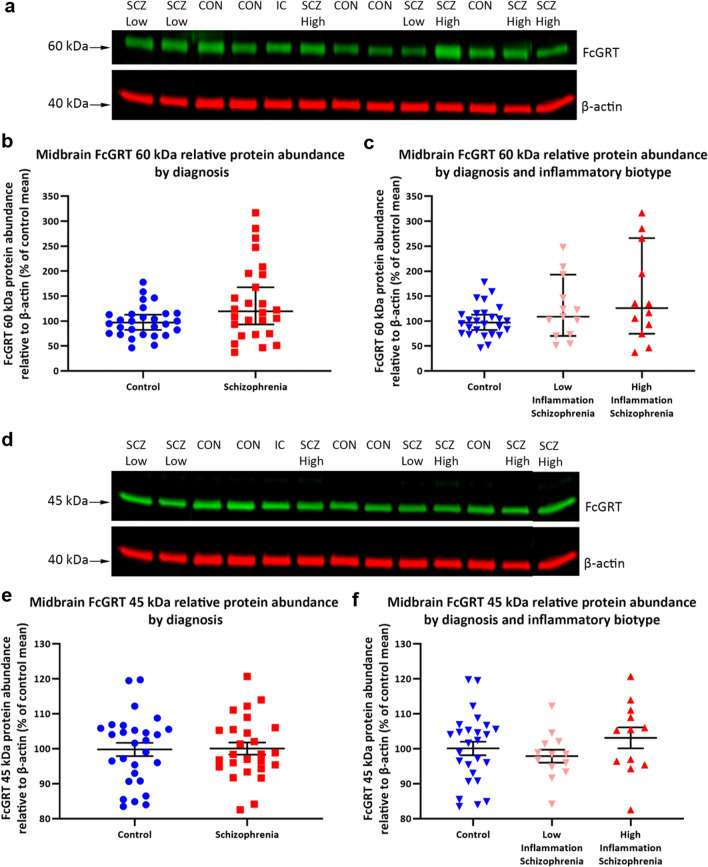
Protein abundance of the IgG transporter, FcGRT, in the midbrain was unchanged when comparing schizophrenia cases and control subjects. FcGRT 60-kDa (**a**) and FcGRT 45-kDa (**d**) immunoreactive bands were normalised to the β-actin band. Both FcGRT 60-kDa and 45-kDa bands were detected in midbrain protein homogenates from all schizophrenia cases and control subjects. FcGRT 60-kDa and FcGRT 45-kDa protein abundance (normalised to β-actin) did not significantly differ when comparing schizophrenia cases and control subjects (**b**, **e**). High-inflammation biotype schizophrenia cases, low-inflammation biotype schizophrenia cases, and control subjects also showed comparable amounts of FcGRT 60-kDa protein and FcGRT 45-kDa protein in the midbrain (**c**, **f**). kDa = kilodalton, SCZ Low = low-inflammation schizophrenia case, SCZ High: high-inflammation schizophrenia case, CON: control subject, IC: internal control. FcGRT 60-kDa is displayed as median ± 95% confidence interval. FcGRT 45-kDa is displayed as mean ± SEM

Two FcGRT-positive immunoreactive bands were detected, one at 60 kDa (Fig. 3a) and one at 45 kDa (Fig. 3d) and were normalised to the β-actin immunoreactive band intensity from the same lane. Comparisons between schizophrenia cases and control subjects revealed no difference for either FcGRT 60 kDa or 40 kDa relative protein abundance (FcGRT 60 kDa: *U* = 476, *p* = 0.075; FcGRT 45 kDa: *t*_(52)_ = − 0.09, *p* = 0.92; Fig. 3b and e). The amount of FcGRT 60-kDa protein also did not significantly differ when comparing high-inflammation biotype schizophrenia cases, low-inflammation biotype schizophrenia cases, and control subjects (*W* = 2.62, *p* = 0.27; Fig. 3c), although the schizophrenia group showed increased variance compared to controls. Similarly, FcGRT 45 kDa relative protein abundance was comparable across the three groups (*F*_(2,48)_ = 0.97, *p* = 0.38; Fig. 3f). Comparable β-actin abundance was found in midbrain from schizophrenia cases and control subjects across the 3 rounds of testing performed (Round 1, *p* = 0.44; Round 2, *p* = 0.59; Round 3, *p* = 0.16).

### FcGRT and FcGR3A transcripts are increased in high-inflammation schizophrenia cases in the midbrain

**Fig. 4:**
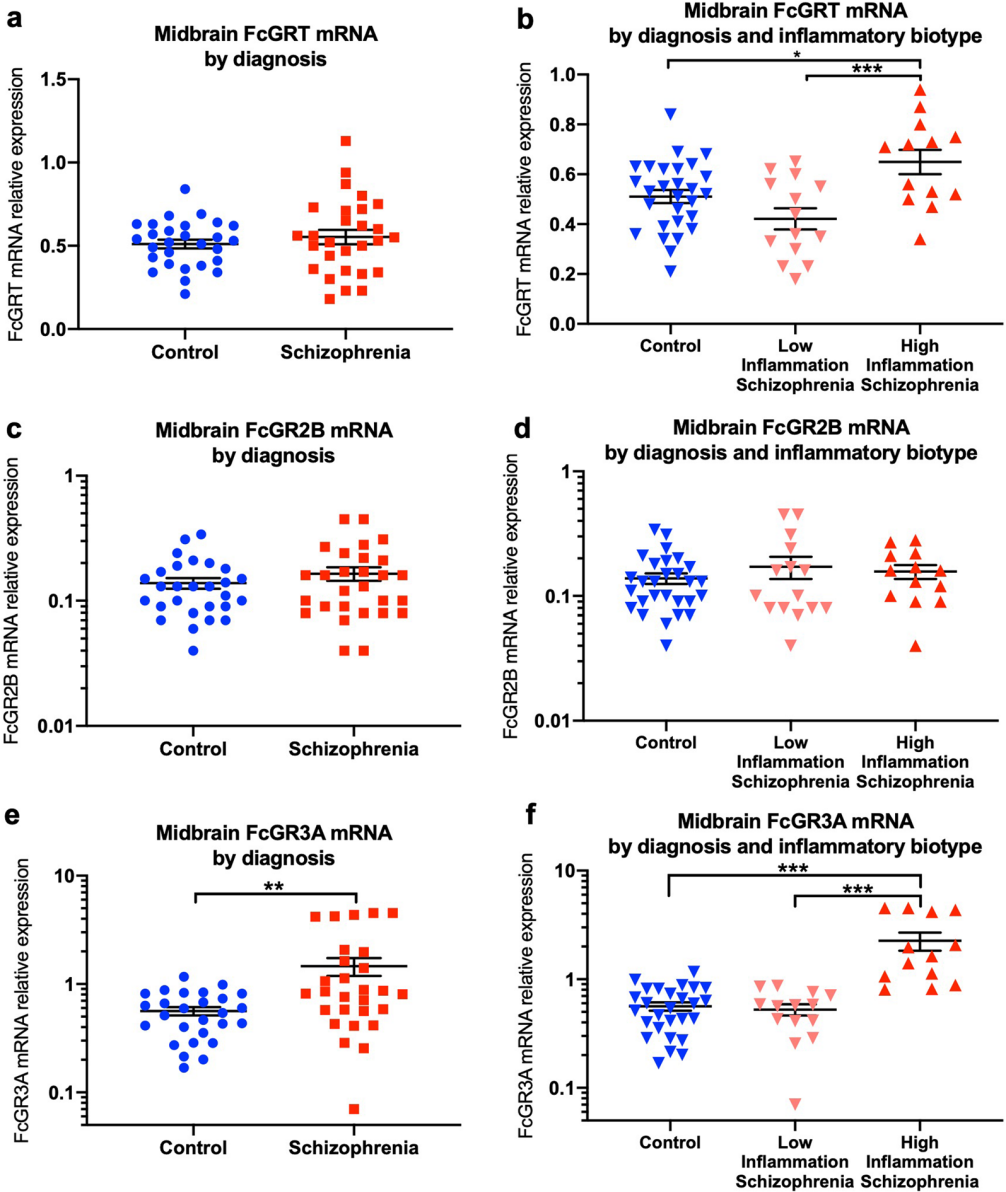
FcGRT and FcGR3A, but not FcGR2B mRNA levels were increased in the midbrain from high-inflammation biotype schizophrenia cases. Midbrain FcGRT mRNA (**a**) and FcGR2B mRNA (**c**) levels were unchanged when comparing schizophrenia cases with control subjects. FcGR3A gene expression was increased in schizophrenia cases compared to control subjects (**e**). When separated by inflammatory biotype, FcGRT mRNA (**b**) and FcGR3A (**f**) mRNA levels were elevated in high-inflammation biotype schizophrenia cases when compared with both the low-inflammation biotype schizophrenia cases and control subjects. There was no difference in FcGR2B mRNA in the midbrain between high-inflammation biotype schizophrenia cases, low-inflammation biotype schizophrenia cases, and control subjects (**d**). *p < 0.05 **p < 0.01 ****p < 0.0001. Mean ± SEM

Gene transcript levels of FcGRT, FcGR3A, and FcGR2B were detected in the midbrain of both schizophrenia cases and control subjects (Fig. 4). FcGRT and FcGR2B mRNA levels in the midbrain from schizophrenia cases were similar to control subjects (FcGRT: *t*_(44.82)_ = 0.82, *p* = 0.41; FcGR2B: *t*_(47.12)_ = 1.07, *p* = 0.28; Fig. 4a and c). However, FcGR3A mRNA was increased by 142.8% in midbrain of schizophrenia cases when compared with control subjects (*t*_(28.79)_ = 3.21, *p* = 0.003; Fig. 4e).

When levels of mRNA were compared between high-inflammation biotype schizophrenia cases, low-inflammation biotype schizophrenia cases, and control subjects, there were overall changes in FcGRT (*F*_(2,52)_ = 7.54, *p* = 0.001; Fig. 4b) and FcGR3A gene expression (*F*_(2,51)_ = 23.83, *p* < 0.001; Fig. 4f). FcGRT mRNA was upregulated by 27.1% in high-inflammation biotype schizophrenia cases compared to control subjects (*p* = 0.02) and by 38.5% compared to low-inflammation biotype schizophrenia cases (*p* < 0.001; Fig. 4b). However, no change was found between low-inflammation biotype schizophrenia cases and control subjects (*p* = 0.18). Similarly, FcGR3A mRNA in midbrain from high-inflammation biotype schizophrenia cases was increased by 275.1% when compared with control subjects (*p* < 0.001) and by 193.0% when compared with low-inflammation biotype schizophrenia cases (*p* < 0.001; Fig. 4f). Low-inflammation schizophrenia cases and control subjects had comparable FcGR3A gene expression in the midbrain (*p* = 0.98). In contrast to the robust up-regulation of FcGRT and FcGR3A, FcGR2B gene expression was not different in the midbrain when comparing high-inflammation biotype schizophrenia cases, low-inflammation biotype schizophrenia cases, and control subjects (*F*_(2,53)_ = 0.65, *p* = 0.52; Fig. 4d).

### 24.1% of all individuals have diffuse halo-like staining of IgG in the SN

**Fig. 5:**
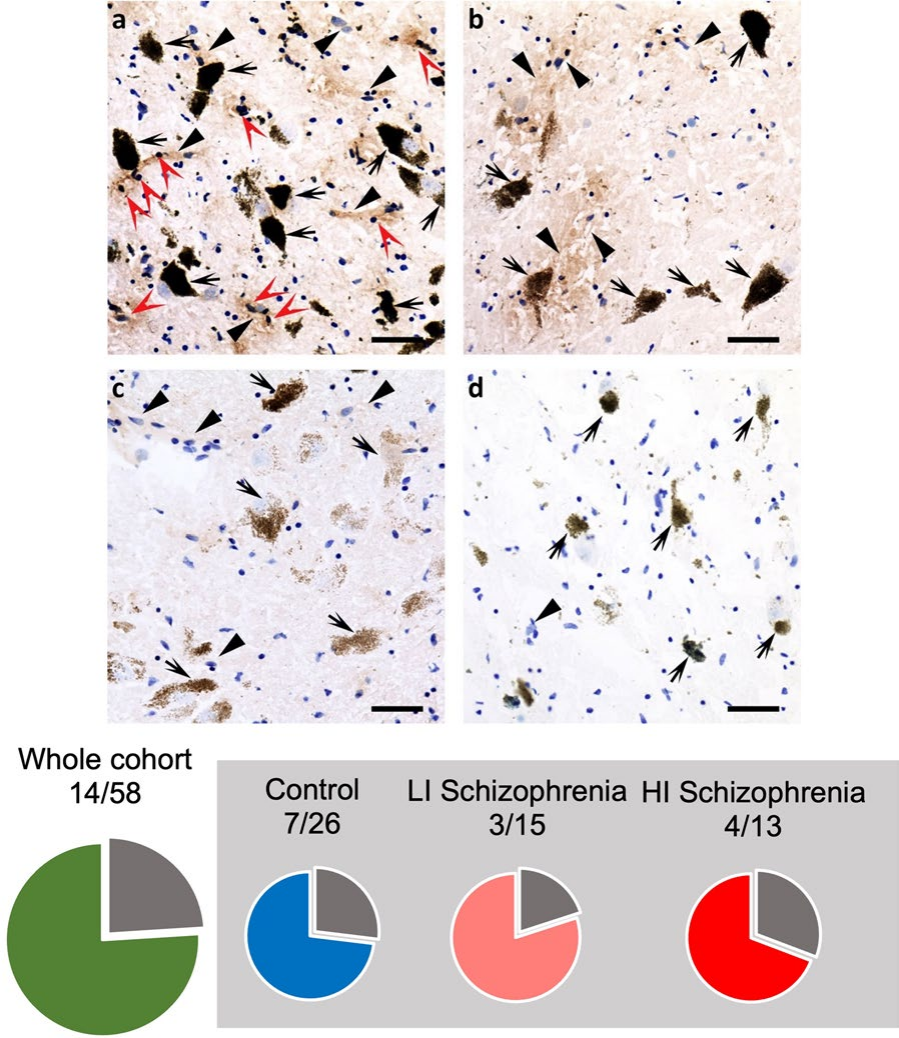
Blood vessels and small cells are IgG positive in the substantia nigra from certain schizophrenia cases and control subjects. Representative images of IgG targeting cells and blood vessels in the substantia nigra (SN) of schizophrenia cases (**a**, **b**) and control subjects (**c**). Dopamine neurons are indicated by black arrows (identified by the presence of neuromelanin). **a** IgG positivity surrounds cells with round nuclei (red arrowheads) adjacent and adhering to IgG positive blood vessels (black closed arrowheads). **b** Diffuse halo of IgG immunoreactivity surrounded IgG positive blood vessels. **c** Faint background staining can be seen in absence of distinctive IgG immunoreactive staining. **d** Immunoreactive staining was absent in no anti-human IgG secondary control slide. Images taken at ×20 magnification. Scale bars are 50 μm

IgG-positive DAB signal product (brown) was found in the SN of schizophrenia cases and control subjects. In 20.7% (12/58) of all individuals, IgG DAB signal product was found to target cells that were adjacent to dopamine neurons (Fig. 5a). These cells had a round dark-blue nucleus consistent with glial-like cells and were surrounded by a halo of IgG immunopositive signal (Fig. 5a). However, the localisation of IgG with these cells in the SN was as likely to occur in schizophrenia cases as for control subjects (*X*^*2*^ = 0.27, *p* = 0.61). We detected a halo of IgG positivity surrounding blood vessels in the SN of 24.1% of individuals (14/58). IgG + cells were frequently found alongside these blood vessels surrounded by a halo of IgG (15.5%, 9/58) (Fig. 5a). However, 8.6% (5/58) of cases had IgG positive blood vessels only (Fig. 5b). The incidence of IgG immunopositive staining surrounding blood vessels in the SN was not significantly different when comparing schizophrenia cases and control subjects (*X*^*2*^ = 0.02, *p* = 0.88). Most individuals (70.7%, 41/58) had neither IgG positive cells nor IgG surrounding blood vessels in the SN (Fig. 5c), however there was a low-level immunopositive signal evident throughout the tissue for all individuals. This is consistent with the data acquired from the western blot analysis where IgG immunoreactive bands were clearly identified in all cases (Fig. 2). The IgG immunoreactive signal in the neuropil was absent from the negative control (a section without the IgG antibody; Fig. 4d), indicating that this is not a background artefact of the DAB staining. Although not quantitatively compared, this pattern of IgG diffusion from blood vessels, as well as the low-level immunopositivity for subjects regardless of diagnosis was also seen in our previous study in the frontal cortex [38].

### Abundance and distribution of IgG in the substantia nigra was unchanged between diagnostic and inflammatory groups

**Fig. 6:**
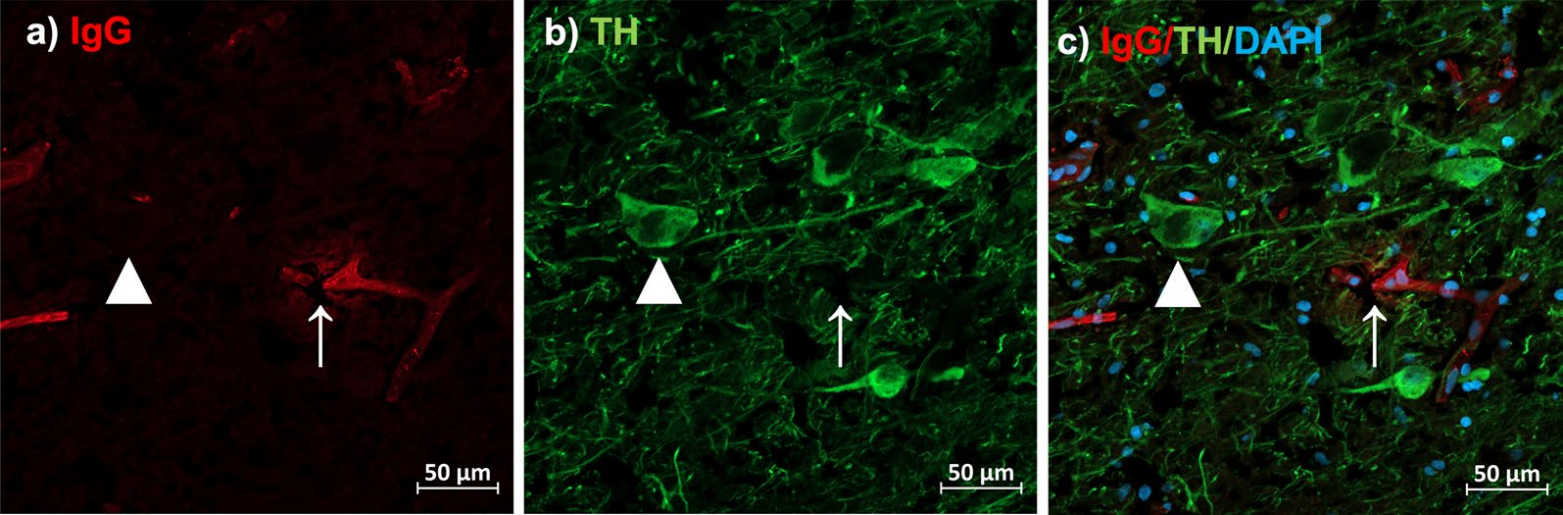
IgG in the SN is distributed throughout blood vessels, as well as within the brain parenchyma, proximal to dopaminergic neurons. In **a**–**c**, the white arrow indicates IgG staining within a blood microvessel and showing diffusion of IgG into the surrounding brain tissue. The white triangle indicates a TH + dopaminergic cell body. Images are at ×20 magnification

**Fig. 7:**
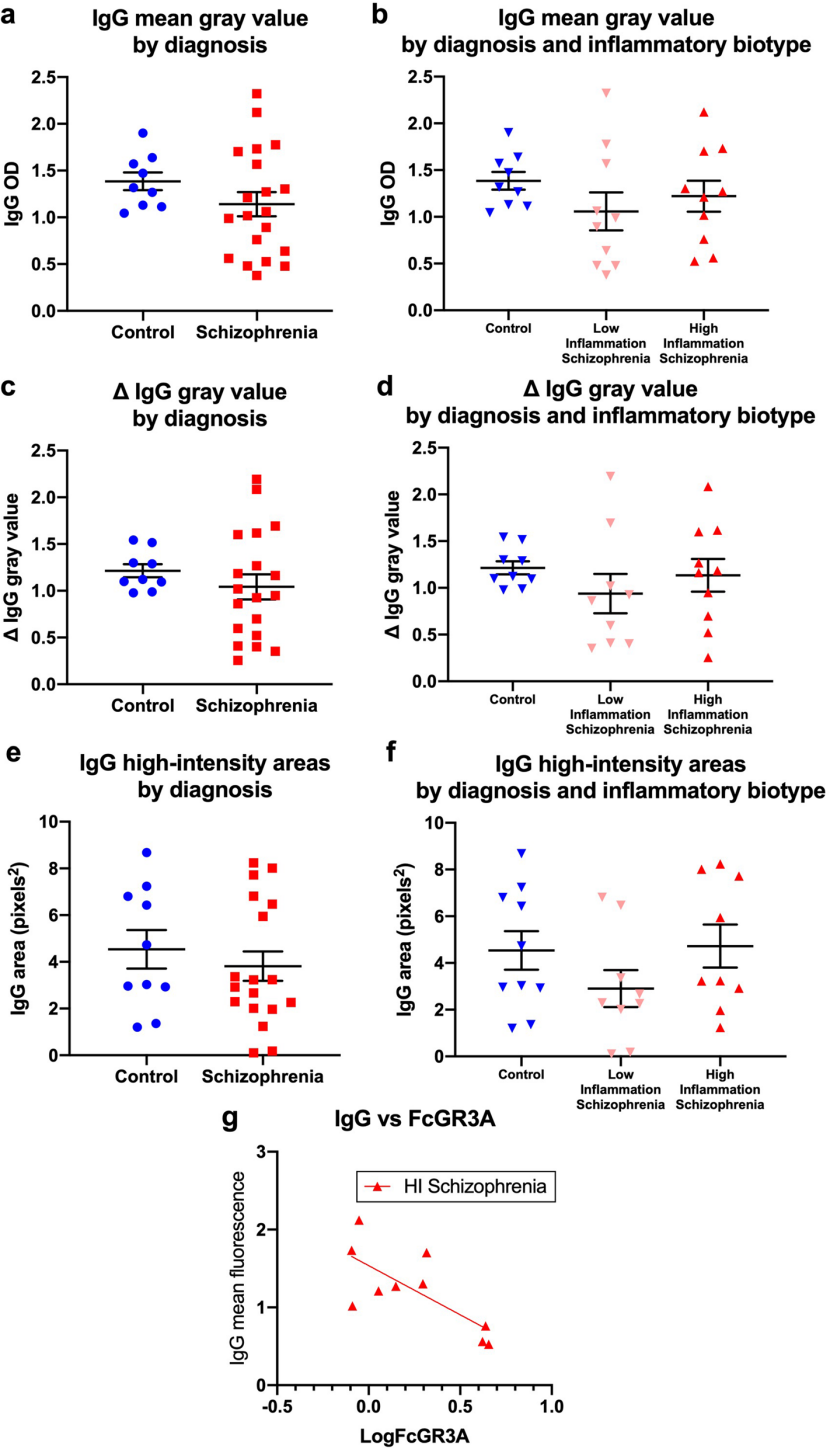
IgG abundance and distribution in the SN is unchanged by either diagnosis or inflammatory biotype. When analysing immunoreactivity (mean gray value) of the IgG channel, there was no difference either **a** by diagnostic group or **b** when schizophrenia subjects were separated in low and high-inflammation biotypes. This was also true when the difference (Δ) in gray values between IgG and no-IgG sections for each individual was analysed (**c**, **d**). Additionally, there was no difference in the total area of high intensity IgG staining (which likely reflects blood vessel and cellular staining as opposed to parenchymal staining) between diagnostic (**e**) or inflammatory groups (**f**). When IgG staining was correlated to LogFcGR3A gene transcript abundance, a significant negative correlation was evident for the high-inflammation biotype schizophrenia group (**g;** *r* = 0.53, *p* = 0.01). OD; optical density. Mean ± SEM

The results seen from the double-labelling immunofluorescence experiment support those seen using DAB staining. IgG was evident in putative blood vessels throughout the SN of both control and schizophrenia subjects (Fig. 6a–c). In some instances, this IgG appeared in a diffuse pattern surrounding blood vessels (Fig. 6a–c, white arrow). There was also evidence of background staining throughout the brain parenchyma, which varied in intensity by individual. However, there was no significant difference in overall IgG immunoreactive intensity between control and schizophrenia patients (*t*_(26.64)_ = 1.53, *p* = 0.13, Fig. 7a). This was also the case when schizophrenia subjects were separated into high- and low-inflammation subtypes (*F*_(2,26)_ = 0.95, *p* = 0.30, Fig. 7b), and when controlling for any background immunofluorescence (Δ gray value; control vs schizophrenia; *t*_(25)_ = 114, *p* = 0.26, control vs low-inflammation vs high-inflammation; *F*_(2,25)_ = 0.71, *p* = 0.49; Fig. 7c, d). When comparing the size of areas of intense IgG staining (with likely staining of blood vessels and cells, as opposed to diffuse parenchymal staining), there was no difference between diagnostic (*U* = 86, *p* = 0.70, Fig. 7e) or inflammatory groups (*F*_(2,26)_ = 0.14, *p* = 0.86, Fig. 7f). However, we found a significant negative correlation between IgG immunoreactive intensity and LogFcGR3A gene transcript values for high-inflammation schizophrenia subjects (*r* = -0.53, *p* = 0.01; Fig. 7g), which was absent for controls (*r* = 0.003, *p* = 0.8) and low-inflammation subjects (*r* = 0.25, *p* = 0.13). To visualise whether any difference in IgG intensity was evident based on anatomical location within the nigra, the IgG intensity of each image was plotted in 2D space for each subject (Additional file 1: Figures S2, S3, S4). No difference in the localization of IgG was apparent between groups. Finally, we found that IgG immunoreactivity was positively correlated with the IgG 50-kDa protein values acquired from the western blot analysis for all subjects (Additional file 1: Figure S5), which supports the validity of fluorescent intensity as a measure of protein abundance in this study.

### Correlations with demographic variables

When analysing the full cohort of individuals, there was a significant negative correlation between pH and FcGRT and FcGR2B mRNA levels (*r* = − 0.26, *p* = 0.05, *r* = − 0.38, *p* = 0.004, respectively). There was also a negative correlation between FcGR2B mRNA and RIN (*r* = − 0.52, *p* < 0.001). However, inclusion of pH or RIN as a covariate did not substantially alter the outcome of group comparisons for these factors (ANCOVA; FcGRT (pH) *F*_(2,52)_ = 3.48, *p* = 0.04; FcGR2B (pH) *F*_(2,52)_ = 0.17, *p* = 0.83; FcGR2B (RIN), *F*_(2,52)_ = 0.89, *p* = 0.41). There were no other correlations between mRNA and age, pH, PMI or RIN, and no significant correlations for any protein outcome (IgG 50 kDa and 25 kDa, FcGRT 63 kDa, 45 kDa) and these factors. There were also no significant correlations between these demographic factors and any of the immunohistochemical outcomes (all *p* > 0.05). When correlating schizophrenia-specific factors (illness onset, illness duration, daily and last dose CPZ), there was a significant positive correlation between LogFcGR3A and daily CPZ dose (*n* = 28, *r* = 0.50, *p* = 0.017), however inclusion of daily CPZ as a covariate did not alter the difference between high- and low-inflammatory schizophrenia biotypes (*F*_(2,19)_ = 6.07, *p* = 0.009). There were no significant correlations between schizophrenia-specific demographics (including the daily and last dose CPZ) and any other mRNA/protein/immunohistochemical outcomes. We also examined correlations between these outcomes and the markers of inflammation used to differentiate high- and low-inflammation schizophrenia subgroups. IgG 50-kDa protein and FcGRT 45-kDa protein abundance were correlated with IL-1B mRNA levels (*p* = 0.04, *p = 0.03*, respectively). There were no other significant correlations between any antibody-related markers assessed and the transcriptional markers of inflammation used to differentiate high- and low-inflammation subgroups.

## Discussion

This study is the first to explore IgG and its receptors in the midbrain of schizophrenia cases and control subjects. Although levels of IgG were unchanged between groups, a novel finding from this work is that people with schizophrenia have a robust increase in gene transcript levels of FcGR3A, a pro-inflammatory IgG receptor, in the midbrain. Furthermore, this effect was driven by the high-inflammation patient group, whereas low-inflammation schizophrenia subjects had expression levels comparable to controls. In contrast, the mRNA levels of the anti-inflammatory receptor FcGR2B were unchanged when comparing schizophrenia cases and control subjects, regardless of the inflammatory biotype of schizophrenia cases. These results suggest that the increase in neuroinflammation seen in the high-inflammation schizophrenia group is not the result of elevated IgG antibody concentrations overall, but an increased potential for effector cell activation driven by IgG. Additionally, this is the first study to show that, in approximately 25% of all individuals, IgG is diffused into the brain parenchyma in the midbrain, likely from the blood vessels which are evident throughout this tissue.

The absence of concurrent FcGR2B up-regulation in the subset of high-inflammation schizophrenia patients suggests an inability to terminate immune complex-Fcγ receptor-mediated processes [39]. Unbalanced expression of activating to inhibitory Fcγ receptors has been implicated in various autoimmune diseases including systemic lupus erythematosus (SLE) and multiple sclerosis (MS) [40–42]. This elevation in FcGR3A may also explain the increase in cytokine levels seen in the same midbrain region of high-inflammation schizophrenia patients. There is evidence that FcGR3A (when bound to IgG immune complexes) results in increased levels of IL-1β, IL-6 and TNFα in T cells isolated from the peripheral blood of SLE patients [43]. Therefore, it is likely that elevated FcGR3A contributes to the pattern of increased levels of IL-1β, IL-6, and TNFα in the midbrain which can be used to define the high-inflammation biotype of schizophrenia [5].

FcGR3A is located on numerous effector cell types throughout the peripheral immune system including macrophages and natural killer cells. Far less is known about its distribution in the brain. However, one study of FcGR3A in the midbrain found that, in healthy people, this receptor was located on cells that were morphologically consistent with lymphocytes [44]. However, the localization of FcGR3A may also be altered by disease state. A previous study which examines the same cohort of schizophrenia and control subjects found an increase in CD163 + cell number in the midbrain that was exaggerated in the high-inflammation schizophrenia group [11]. It is therefore possible that changes seen in the current study do not reflect more FcGR3A per cell, but rather an increase in FcGR3A-expressing macrophages. An interaction between these FcGR3A-expressing macrophages and IgG may contribute to the increase in complement C1q transcripts, also seen in the midbrain of high-inflammation patients [11]. Further work to clarify the cell-specific localization of FcGR3A in the midbrain of the high-inflammation subgroup is necessary to support this hypothesis.

Interestingly, we found that the elevated levels of FcGR3A in the high-inflammation schizophrenia patients were negatively correlated with IgG levels in the midbrain. Phagocytosis, antibody-dependent cellular cytotoxicity (ADCC), and antigen presentation induced by immune complex-FcGR3A binding results in lysosomal degradation of IgG along with the antigen to which it is bound. This may account for the inverse relationship between FcGR3A and IgG. However, caution is required for interpreting this result since it was a correlation between transcript levels for FcGR3A and protein levels for IgG. Exploration of FcGR3A and B cell activities would better determine the impact of IgG production and degradation in the schizophrenia midbrain.

Although levels of FcGRT protein were unchanged between control subjects and schizophrenia patients (either high- or low-inflammation biotypes), we found that FcGRT mRNA was increased in the midbrain from schizophrenia cases with the high-inflammation biotype. As such, we propose that FcGRT protein turnover in the midbrain is increased in high-inflammation biotype schizophrenia cases. FcGRT rescues bound IgG from lysosomal degradation by recycling it back to the cell surface via sorting endosomes [45]. This mechanism enables FcGRT to move IgG from the brain into the blood in a unidirectional manner [46]. Given that levels of the IgG protein in the midbrain were not different between diagnostic groups, this may suggest that the synthesis of IgG is elevated in the brain of high-inflammation patients, but this is balanced by increased export from the brain into the blood via the FcGRT, such that there is higher turnover or trafficking of IgG. Such a proposal could be investigated by determining the subcellular location of IgG in midbrain blood vessels the midbrain from high-inflammation schizophrenia patients.

There is evidence of increased dopaminergic activity in the midbrain of people with schizophrenia [36, 47, 48]. However, it is unknown whether the elevation of FcGR3A gene transcripts seen in the high-inflammation schizophrenia subgroup is related to this hyperdopaminergia. Indeed, there is evidence that dopamine can activate immune cells. For example, dopamine increases monocyte migration into the brain and induces the release of IL-6 from monocyte-derived macrophages [15, 49]. Additionally, microglia stimulated to uptake dopamine by membrane dopamine transporter are primed towards phagocytosis as revealed by decreased processes and increased mitogen-activated protein kinase pathway activation [50]. Dopamine receptors are also found on both microglia [50, 51] and astrocytes in the brain [52, 53]. Therefore, increased dopaminergic activity in the midbrain of schizophrenia patients may stimulate an immune response in the brain, which might contribute to increased levels of FcGR3A. However, the same pattern of findings in the midbrain has also been found in the prefrontal cortex; levels of IgG in the brain were unaltered [38], and levels of FcGR3A were increased only in the high-inflammation schizophrenia subgroup [16] in this region. This may suggest that local hyperdopaminergia is not a primary contributor to FcGR3A alterations in the midbrain in these patients, since cortical regions contain only sparse dopaminergic innervation in comparison to the midbrain, and there is evidence of reduced dopaminergic activity in these regions in people with schizophrenia [54–56]. Further work in animal models of nigral hyperdopaminergia is therefore necessary to clarify the relationships between elevated dopaminergic activity in the midbrain and neuroinflammation.

One limitation of this work is that this these techniques to assess IgG abundance do not differentiate between specific IgG types. Although the levels of antibodies are unchanged between groups, we have no information about the antigens that these antibodies may target, or whether they are bound or unbound to an antigen. Antibodies will only bind to IgG receptors if they have formed an immune complex by attaching to an antigen. It is therefore possible that, in the high-inflammation subgroup, an increase in the number of immune complexes (IgG bound to an antigen) is stimulating an increase in the pro-inflammatory FcGR3A receptor. Although these antigens can be foreign to the body (eg. bacterial or viral pathogens), there are instances of autoantibodies (antibodies generated against an individual’s own proteins) contributing to schizophrenia-like symptoms. For instance, in anti-N-methyl-D-aspartate (NMDA) receptor encephalitis, the generation of antibodies against the NMDA receptor results in NMDA hypofunction and schizophrenia-like symptoms including psychosis [57]. Therefore, pathologically relevant autoantibodies in schizophrenia may promote the expression of pro-inflammatory Fcγ receptors within in the midbrain. An additional limitation to this study is that we have not clarified the source of IgG in the brain. Future studies will examine the abundance of B cells in the brain, to determine whether the IgG identified in this work is generated locally in the brain, or transported into the brain parenchyma through the microvasculature.

## Conclusion

Taken together, these results demonstrate that the immune dysfunction of a subset of schizophrenia cases extends to the adaptive immune system. We found the high-inflammation biotype schizophrenia cases demonstrated increased expression of both the IgG transporter, FcGRT, and one of the pro-inflammatory receptors, FcGR3A. Yet the inhibitory receptor, FcGR2B, was unchanged, suggesting a more volatile antibody-driven response in some individuals with schizophrenia. While IgG was evident in the midbrain of all individuals, the abundance and overall distribution of IgG was unchanged by diagnostic or inflammatory group. We speculate that IgG engaging with FcGR3A on phagocytic cells, such as microglia or macrophages, contributes to elevated cytokines in midbrain from high-inflammation biotype schizophrenia cases. As well as further supporting the hypothesis of a neuroinflammatory component to schizophrenia, this study reinforces the need to cluster patients into high- and low-inflammation subtypes. Understanding these subtypes may reveal significant differences in underlying neurobiology which would be pertinent to developing efficacious treatments. Finally, this study revealed that IgG diffuses into the midbrain from blood vessels in both healthy people and in patients with schizophrenia. This study therefore contributes to our understanding of the distribution and function of IgG and its receptors in both the healthy brain and in disease states.

## Supplementary Information

**Additional file 1.** Supplementary methods and figures.

**Additional file 2.** Detailed demographics for complete cohort.

**Additional file 3.** Western blot raw figures.

## Data Availability

The datasets used and/or analysed during the current study are available from the corresponding author on reasonable request.

## Abbreviations

IgG: Immunoglobulin-G
FcGRt: Fc gamma receptor and transporter
FcGR3A: Immunoglobulin gamma Fc region receptor IIIa
FcGR2B: Immunoglobulin gamma Fc region receptor IIb
ICAM: Intercellular adhesion molecule 1
ITAM: Immunoreceptor tyrosine-based activation motif
ITIM: Immunoreceptor tyrosine-based inhibitory motif
DAB: 3,3′-Diaminobenzidine
IL-6: Interleukin-6
IL-1β: Interleukin-1β
SERPINA3: Serpin peptidase inhibitor clade A member 3
TNFα: Tumour necrosis factor-α
RIN: RNA integrity number
CPZ: Chlorpromazine
PMI: Post-mortem interval
PVDF: Polyvinylidene fluoride
TBS: Tris-buffered saline
RT: Room temperature
ABC: Avidin–biotin-peroxidase complex
TH: Tyrosine hydroxylase
DAPI: 4′,6-Diamidino-2-phenylindole
ANOVA: Analysis of variance
ANCOVA: Analysis of covariance
kDa: Kilodalton
IC: Internal control
SN: Substantia nigra
OD: Optical density
SLE: Systemic lupus erythematosus
MS: Multiple sclerosis
ADCC: Antibody-dependent cellular cytotoxicity
NMDA: *n*-Methyl-d-aspartate
