# Supplementary material for: Increased levels of a pro-inflammatory IgG receptor in the midbrain of people with schizophrenia
Source: J Neuroinflammation. 2022 Jul 15;19:188. doi: 10.1186/s12974-022-02541-8 (PMC9287858; doi:10.1186/s12974-022-02541-8)
Supplement: Supplementary file 1 — Additional file 1. Supplementary methods and figures. [file 12974_2022_2541_MOESM1_ESM.docx]

Additional information:

**Additional file 1: Table S1:** Demographic and clinical variables comparing control subjects with either all schizophrenia cases, or control subjects compared to both the low inflammation and high inflammation schizophrenia biotype groups, for the immunofluorescence analysis.

|  | **Immunofluorescence Cohort** | | | | | |
| --- | --- | --- | --- | --- | --- | --- |
| Demographics | Control | Schizophrenia | Statistics | Schizophrenia (low inflammation) | Schizophrenia (high inflammation) | Statistics |
| n | 10 | 20 |  | 10 | 10 |  |
| Age (years) | 53.5 (33-63) | 56.0  (26-67) | *t*_(28)_=-0.73, *p*=0.47 | 47.5  (37-64) | 57.5  (26-67) | *F*_(2, 27)_=1.34, *p*=0.27 |
| Sex (M,F) | 6,4 | 12,8 | *X^2^*=0.00, *p*=1.0 | 6,4 | 6,4 | *X^2^*=0.00, *p*=1.0 |
| pH | 6.5 ± 0.25 (6.1-6.8) | 6.5 ± 0.1  (6.1-6.8) | *t*_(28)_=0.38 *p*=0.07 | 6.5 ± 0.2  (6.1-6.8) | 6.5 ± 0.1  (6.2 – 6.8) | *F*_(2,27)_=0.13, *p*=0.89 |
| PMI (h) | 35.35 ± 8.81 (24-50) | 35.38 ± 19.04 (5-72) | *t*_(28)_=0.003, *p*=0.99 | 31.1 ± 13.4 (18-49) | 39.6 ± 23.3  (5-72) | *F*_(2,27)_=0.67, *p*=0.52 |
| Age at illness onset (years) | - | 21.9 ± 6.9  (15-46) | – | 18.8 ± 2.6  (15-22) | 25.0 ± 8.5  (16-46) | ***U*=22, *p*=0.03*** |
| Duration of illness (years) | - | 31.00 ± 12.55 (5-49) | – | 30.3 ± 9.2  (16-43) | 31.7 ± 15.6  (5-49) | *t*_(18)_=024, *p*=0.81 |
| Daily CPZ equivalent dose (mg)^a^ | - | 675.2 ± 426 (250-1850) | – | 461.8 ± 152.1  (350-800) | 919.1 ± 516.1 (250-1850) | ***U*=11.0, *p*=0.05*** |
| Last recorded CPZ equivalent dose (mg)^b^ | - | 593.6 ± 502.0 (20-1732) | – | 335.0 ± 184.3 (20-580) | 852.2 ± 591.2  (100-1732) | ***U*=22.5, *p*=0.03^*^** |

Age is written as median (range). All other values are written as mean ± standard deviation (range). M=male, F=female; PMI=post-mortem interval; CPZ=chlorpromazine.

**Additional file 1: Table S3:** List of TaqMan Assays used to probe for Fc γ receptors in the midbrain from schizophrenia cases and control subjects

| *Fcγ receptors* | | |
| --- | --- | --- |
| Gene symbol | Gene name | TaqMan assay |
| FcGRT | Fc γ receptor transporter | Hs01108967 |
| FcGR2B | Fc γ receptor 2B | Hs00269610 |
| FcGR3A | Fc γ receptor 3A | Hs02388314 |

**Additional file 1: Table S4:** Outliers removed for each outcome, separated by diagnostic/inflammatory group.

| ***Outliers/group*** | ***Control*** | ***LI Schizophrenia*** | ***HI Schizophrenia*** |
| --- | --- | --- | --- |
| IgG 50kDa protein | 0 | 1 | 0 |
| IgG 25kDa protein | 1 | 1 | 0 |
| FcGRT 60kDa protein | 0 | 0 | 0 |
| FcGRT 45kDa protein | 0 | 0 | 0 |
| FcGRT mRNA | 0 | 1 | 0 |
| FcGR2B mRNA | 0 | 0 | 0 |
| FcGR3A mRNA | 1 | 1 | 0 |
| IgG mean gray value | 1 | 0 | 0 |
| IgG delta gray value | 1 | 0 | 0 |
| IgG high-intensity areas | 0 | 1 | 1 |

LI: low inflammation. HI: high inflammation.

Additional figures:

**Pipeline for quantifying IgG particles from immunofluorescent experiment:**


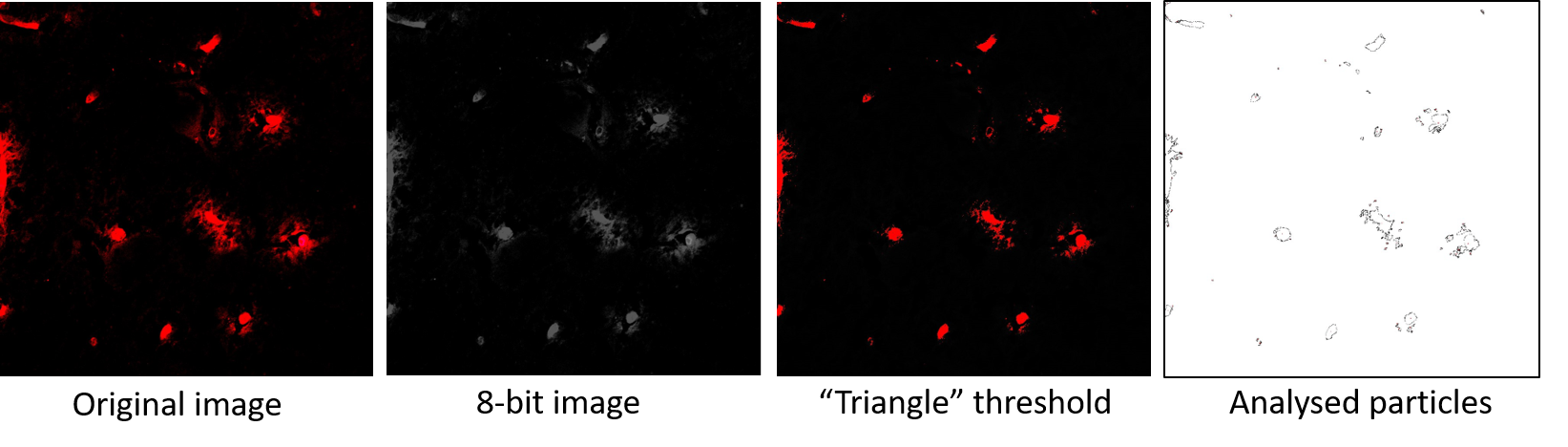


**Additional file 1: Figure S1.** The original single channel image (red channel = IgG) was converted to an 8-bit image first. Then, the “Triangle” threshold was applied (included in the ImageJ software) to remove lower intensity (primarily background) staining, and leave the regions of high intensity, which likely reflect blood vessels, and cellular staining. “Analyse particles” was applied to quantify these regions and provide an area of each particle.


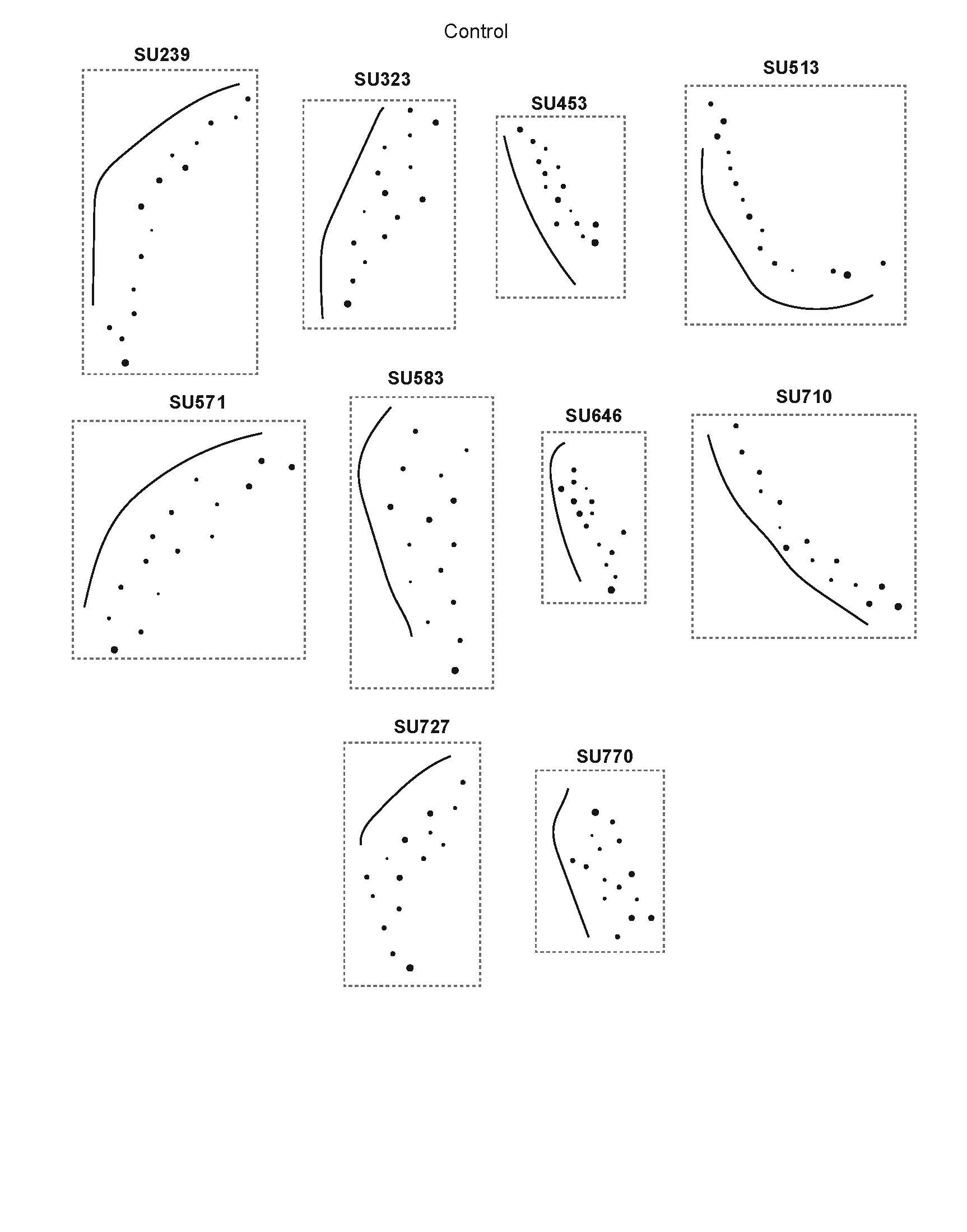


**Additional file 1: Figure S2.** Anatomical position of each of the 15 snaps per subject for the control group. Each circle represents the IgG of each snap, with the size of the circle representing the amount of IgG present. The solid line indicates where the cerebral peduncles sit in relation to the substantia nigra.


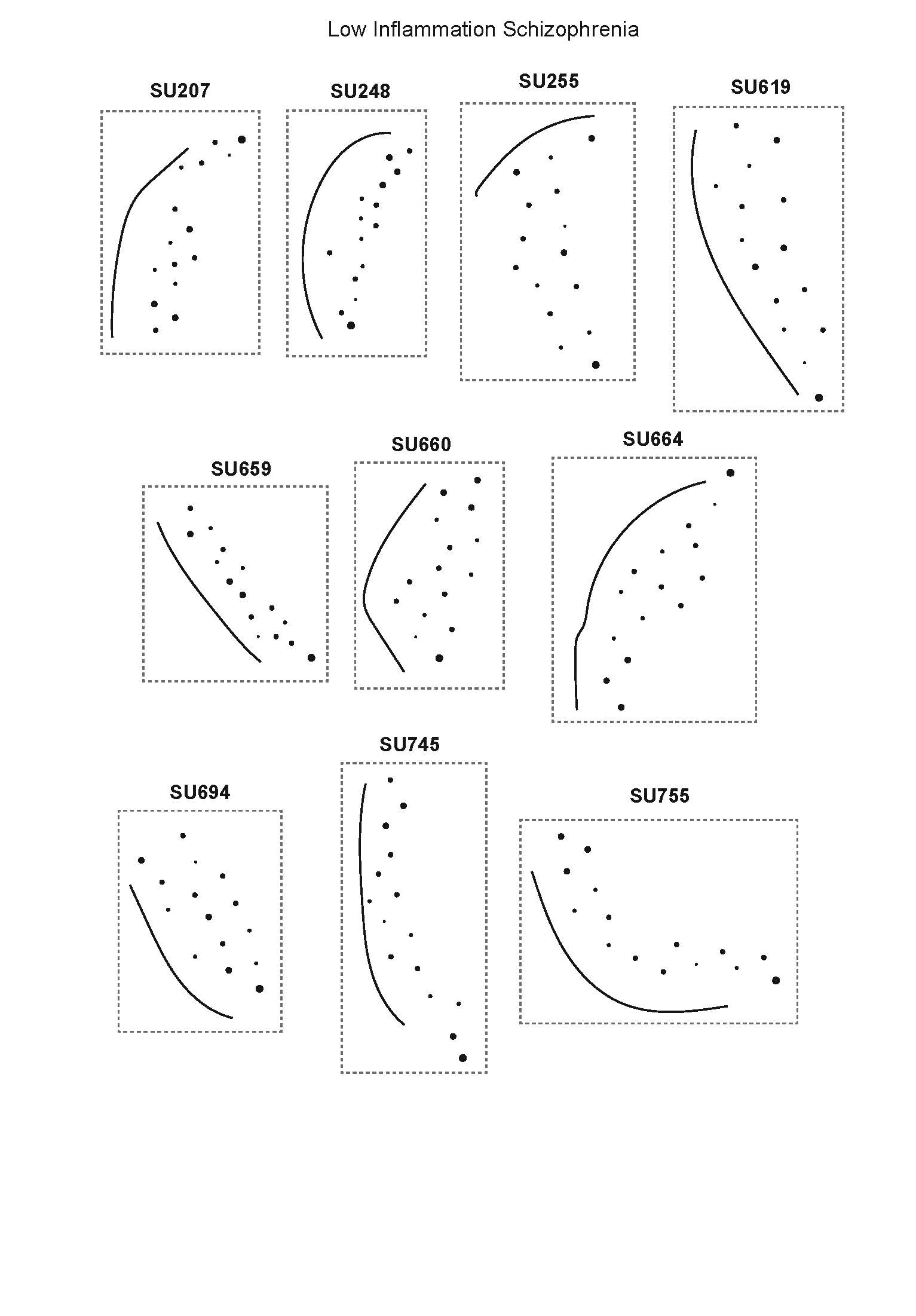


**Additional file 1: Figure S3.** Anatomical position of each of the 15 snaps per subject for the low inflammation schizophrenia group. Each circle represents the IgG of each snap, with the size of the circle representing the amount of IgG present. The solid line indicates where the cerebral peduncles sit in relation to the substantia nigra.


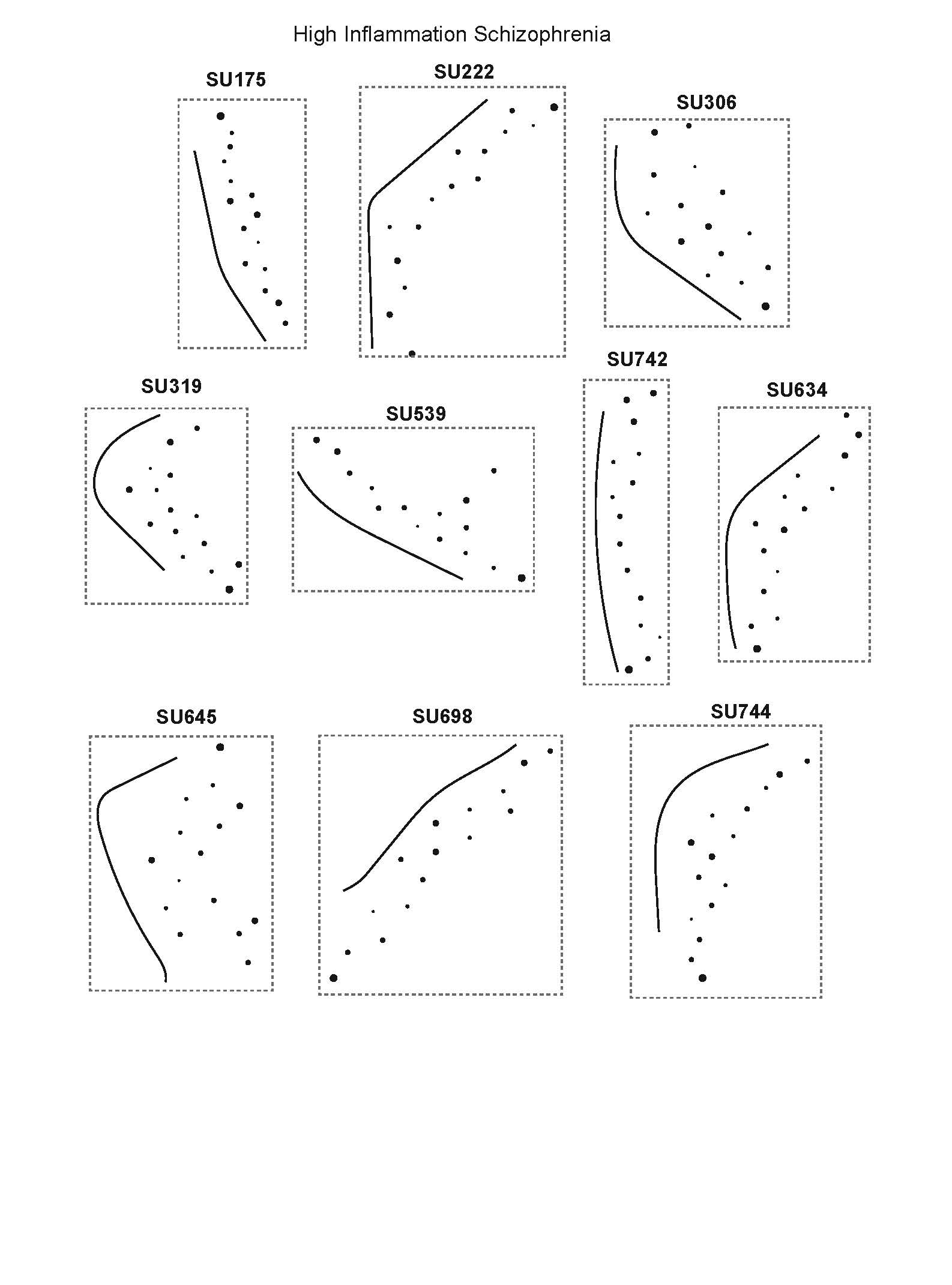


**Additional file 1: Figure S4.** Anatomical position of each of the 15 snaps per subject for the high inflammation schizophrenia group. Each circle represents the IgG of each snap, with the size of the circle representing the amount of IgG present. The solid line indicates where the cerebral peduncles sit in relation to the substantia nigra.


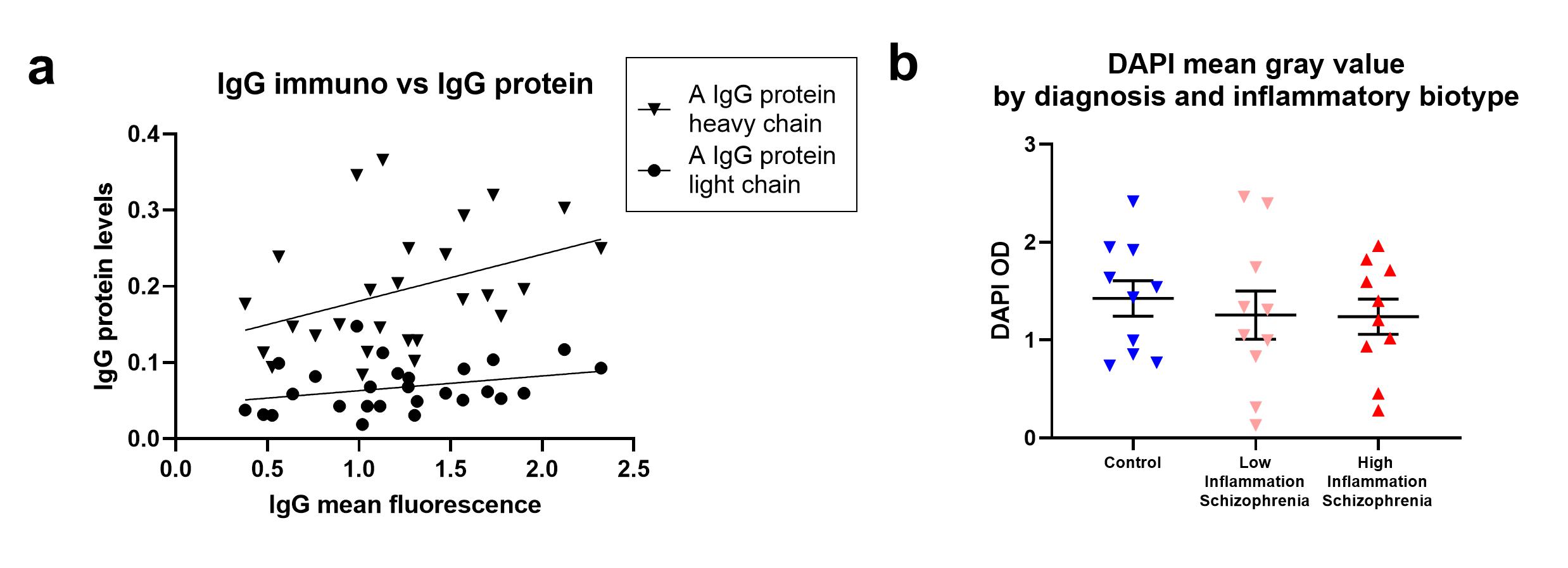


**Additional file 1: Figure S5.** Relationship between IgG measured in two difference techniques. A positive correlation was found between IgG mean fluorescence and the IgG protein abundance as measured by western blot, although this was only the case for the IgG heavy chain protein (*R*=0.15, *p*=0.04), but not the light chain (*R*=0.09, *p*=0.1).
